# Novel Genes and Metabolite Trends in *Bifidobacterium longum* subsp. *infantis* Bi-26 Metabolism of Human Milk Oligosaccharide 2′-fucosyllactose

**DOI:** 10.1038/s41598-019-43780-9

**Published:** 2019-05-28

**Authors:** Bryan Zabel, Christian Clement Yde, Paige Roos, Jørn Marcussen, Henrik Max Jensen, Krista Salli, Johanna Hirvonen, Arthur C. Ouwehand, Wesley Morovic

**Affiliations:** 1 Genomics & Microbiome Science, DuPont Nutrition & Health, Madison, WI USA; 2 Advanced Analysis, DuPont Nutrition Biosciences ApS, Brabrand, Aarhus, Denmark; 3 Genomics Lab, DuPont Pioneer, Johnston, IA USA; 4 Global Health and Nutrition Science, DuPont Nutrition and Health, Kantvik, Finland

**Keywords:** Applied microbiology, Industrial microbiology

## Abstract

Human milk oligosaccharides (HMOs) function as prebiotics for beneficial bacteria in the developing gut, often dominated by *Bifidobacterium* spp. To understand the relationship between bifidobacteria utilizing HMOs and how the metabolites that are produced could affect the host, we analyzed the metabolism of HMO 2′-fucosyllactose (2′-FL) in *Bifidobacterium longum* subsp. *infantis* Bi-26. RNA-seq and metabolite analysis (NMR/GCMS) was performed on samples at early (A600 = 0.25), mid-log (0.5–0.7) and late-log phases (1.0–2.0) of growth. Transcriptomic analysis revealed many gene clusters including three novel ABC-type sugar transport clusters to be upregulated in Bi-26 involved in processing of 2′-FL along with metabolism of its monomers glucose, fucose and galactose. Metabolite data confirmed the production of formate, acetate, 1,2-propanediol, lactate and cleaving of fucose from 2′-FL. The formation of acetate, formate, and lactate showed how the cell uses metabolites during fermentation to produce higher levels of ATP (mid-log compared to other stages) or generate cofactors to balance redox. We concluded that 2′-FL metabolism is a complex process involving multiple gene clusters, that produce a more diverse metabolite profile compared to lactose. These results provide valuable insight on the mode-of-action of 2′-FL utilization by *Bifidobacterium longum* subsp. *infantis* Bi-26.

## Introduction

A large proportion of ingested human milk oligosaccharides (HMOs) reach the large intestine in an intact form and act as prebiotics by providing a metabolic substrate for the growth and activity of potentially beneficial gut bacteria, such as *Bifidobacterium* species^1–3^. High levels of *Bifidobacterium* spp. are important as infant onset intestinal dysbiosis and lower number of *Bifidobacterium* spp. has been clinically shown to predispose infants to inflammation, increase risks for obesity, allergies, inflammatory bowel disease and diabetes^4^. Thus, HMOs impact the development of the early microbiota of the infant. In addition to the prebiotic function, accumulating evidence supports the role of HMOs in promoting gut, immune and cognitive health in infants^5,6^.

HMOs are the third most abundant solid component in human milk after lactose and lipids^7^. They consist of more than 200 structurally diverse carbohydrate polymers that are composed of five monomers: D-glucose, D-galactose, *N*-acetylglucosamine, L-fucose and *N*-acetylneuraminic acid (sialic acid). All HMOs contain lactose at their reducing end which can be fucosylated with the simplest form being 2′- or 3-fucosyllactose (2′-FL or 3FL), or sialylated with the simplest form being 3′- or 6′-sialyllactose (3′-SL or 6′-SL). The concentration of HMOs in mature milk is estimated to be ~10–15 g/L, with 2′-FL being the most abundant^5,8^. However, the HMO concentrations vary between individuals and during lactation^9^. Recent advances in large-scale production of individual HMOs enable the supplementation of these oligosaccharides to infant formula^10,11^.

*In vitro* experiments have revealed strain-dependent utilization of individual HMOs among *Bifidobacterium*, *Lactobacillus* and *Bacteroides* species^12–19^. Infant *Bifidobacterium* species, such as *Bifidobacterium longum* subsp. *infantis* and *Bifidobacterium bifidum*, possess enzymes including α-fucosidases, sialidases, and β-galactosidases that are required for metabolism of HMOs. However, these species also have unique strategies to utilize HMOs. *B. infantis* degrades and utilizes intact HMOs completely intracellularly while *B. bifidum* can only utilize the glucose and galactose portions of HMOs while exporting fucose^12,17,18,20,21^. *B. infantis* is unique among gut bacteria in its high capacity to consume a wide variety of HMO structures by expressing several ABC transporters and glycosyl hydrolases encoded in a HMO utilization gene cluster broadly conserved through *B. infantis* strains^12^.

Pathways of HMO metabolism have previously been explored genomically and by comparative transcriptomics in infant-associated *Bifidobacterium* species^14–16^. However, little has been explored on the connection between specific genes involved in specific monomers of HMO (2′-FL) consumption and the resulting metabolites. Therefore, we analyzed the metabolism of 2′-FL compared to lactose, the reducing end of 2′-FL, by *B. longum* subsp. *infantis* Bi-26 (Bi-26) using comparative genomics, RNA-seq, and metabolite analysis. Bi-26 is a commercially available probiotic, isolated from infant feces, and hypothesized to grow on 2′-FL because of subspecies identity. The genome and gene expression of Bi-26 were also compared to those identified in previous experiments using *B. infantis* type strain ATCC 15697 (ATCC 15697). Through transcriptomic and metabolite analysis we can better understand the utilization of 2′-FL by *B. longum* subsp. *infantis* Bi-26 strain, and thus demonstrate the potential synergistic function of this HMO-probiotic combination.

## Results

### Genome features of *B. longum* subsp. *infantis* Bi-26

The draft genome of Bi-26 sequence contains 2,569,657 base pairs (bp) over 41 contiguous sequences (contigs) that code for 2280 genes and 60 noncoding RNA genes. The GC content is 59.4% which is consistent with other strains in this subspecies. The assembly resulted in a weighted median contig statistic (N50) score of 143,137 with the smallest number of contigs whose length sum produces N50 (L50) score of 6. Those 2280 genes translate into 1128 functions (Supplementary Table 1). The genome contains the necessary enzymes to breakdown 2′-FL into its monomers (fucose, glucose, lactose) along with the Leloir pathway, fucose, and central metabolism genes to potentially process the monomers into 1,2-propanediol (1,2-PD), lactate and acetate as described in Supplementary Table 2 ^17,18^.

### Comparative genomics of *B. longum* subsp. *infantis* Bi-26

**Figure 1:**
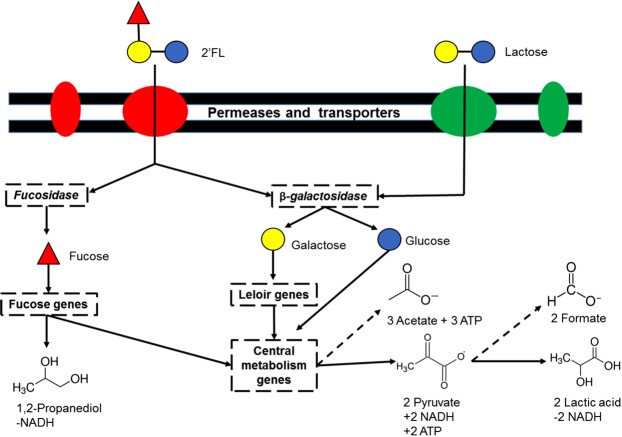
Pathway of 2′-FL and lactose utilization by *Bifidobacterium longum* subsp. *infantis* Bi-26. Dashed boxes have genes for the specific functions located in Table S2. Dashed lines show potential of different metabolites coming from same intermediate compound. Quantified metabolites are also shown.

Bi-26 was compared to the previously sequenced ATCC 15697^22,23^. Overall, Bi-26 and ATCC 15697 have an average nucleotide identity of 98.2% (81.2% coverage). Bi-26 share 967 functional roles with ATCC 15697 with 131 unique functions for Bi-26 and 36 for ATCC 15697 (Supplementary Table 1). The unique functions of Bi-26 include utilization of various carbon sources like maltose, maltodextrin, xylose, and inositol, extracellular peptides, CRISPR genes, transporters, and prophage elements. The previously identified 43kbp HMO utilization cluster in ATCC 15697^12^ was in Bi-26 with 25 out of the 29 genes matching (Supplementary Fig. 1). The insertion sequence (IS) element that was previously described in ATCC 15697 was not present in Bi-26 along with two transport genes and one solute binding protein gene. This results in an HMO cluster consisting of 35.6 kb with an 82.2% base pair identity to ATCC 15697. Both strains, along with 7 other *B. longum* subsp. *infantis* strains tested, share the same Leloir and fermentative pathway genes necessary to breakdown glucose and galactose through the bifid shunt (Supplementary Table 2)^24^. The bifid shunt allows bifidobacteria to break down monosaccharides to short chain fatty acids (SCFAs) without using glycolysis^24^. Bifidobacteria lack the genes necessary to utilize the glycolysis pathway and therefore use the fructose-6-phosphoketolase enzyme to produce more energy from various carbon sources. Fucose utilizing genes are conserved in all tested *B. longum* subsp. *infantis* genomes, all having the necessary genes to produce 1,2-PD (Supplementary Table 2). The main products of both 2′-FL and lactose fermentation can be predicted as acetate, formate, pyruvate, lactate, and 1,2-PD in 2′-FL fermentation (Fig. 1).

### Growth of *B. longum* subsp. *infantis* Bi-26 on 2′-FL, and lactose

**Figure 2:**
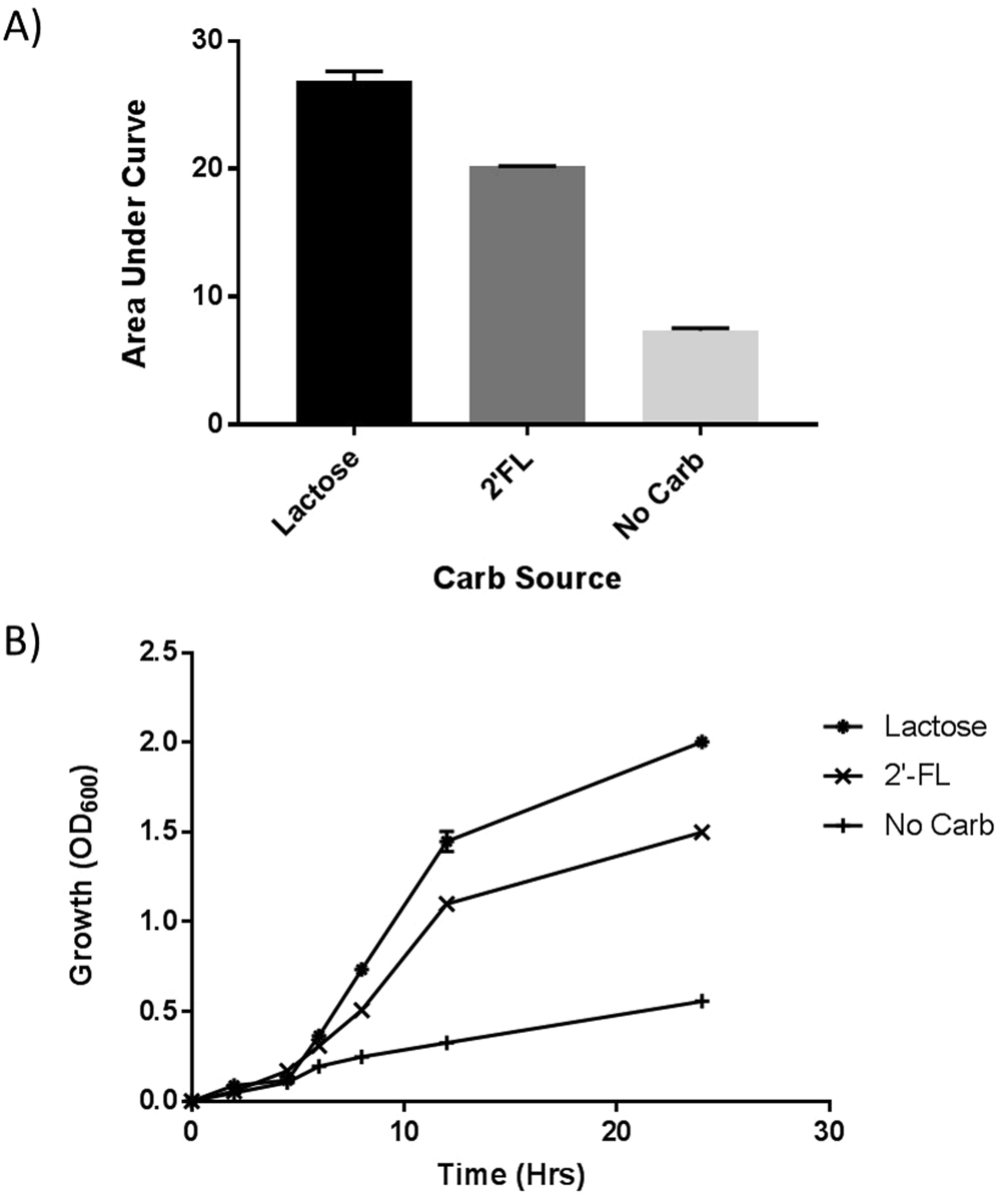
Growth curves of *Bifidobacterium longum* subsp. *infantis* Bi-26 strain grown with various carbon sources. (**A**) Growth curves (A600) of *Bifidobacterium longum* subsp. *infantis* Bi-26 strain grown with 2′-FL, Lactose, and No carbohydrate added in mBM58 media. (**B**) Area under the curve data for the growth curves from (**A**) *Bifidobacterium longum* subsp. *infantis* Bi-26 grown on 2′-FL, Lactose, and No carbohydrate. A600 absorbance was set to zero after inoculation.

Figures 2A,B show the growth curves of Bi-26 on various carbon sources including 2′-FL, lactose, and no carbohydrate added as the controls. The samples in lactose had fast growth and reached a max A600 of 2.0 in 24 hours. Samples of 2′-FL was slower and reached a max A600 of 1.5 in 24 hours while the no carbohydrate control reached 0.75 in 24 hours. Growth on lactose resulted in the largest area under the growth curve (AUC) (100%) with 2′-FL at 73.5% of the AUC, and no carbohydrate at 26.3% of the AUC in a 24-hour growth.

### Transcriptome of *B. longum* subsp. *infantis* Bi-26 in response to 2′-FL utilization

**Figure 3:**
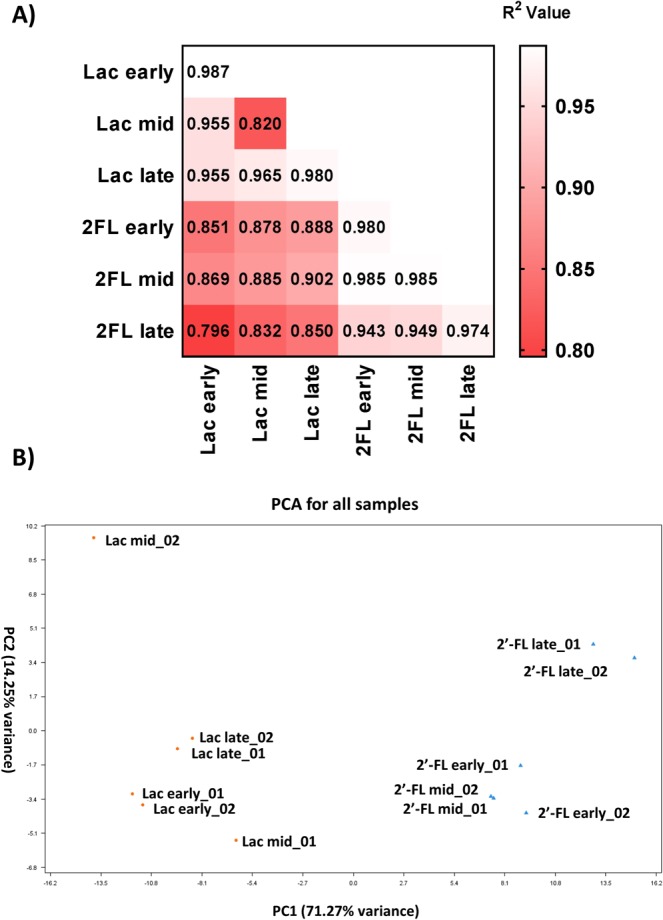
(**A**) Comparisons of individual samples taken R^2^ value with the comparison of the individual replicates for each sample type down the middle. (**B**) PCA plot of all individual samples together to compare their likeness.

A minimum of 10,721,263 reads for each sample from early (A600 0.25–0.30) mid-log (0.5–0.7) and late-log (1.0–2.0) phases throughout the 2′-FL fermentation were assembled to the Bi-26 draft genome. Transcriptomes were compared using principal component analysis (PCA) resulting in lactose and 2′-FL samples clustering separately along PC1 (Fig. 3B). 2′-FL early phase transcriptomes and mid-log phase transcriptomes clustered near to each other with late-log phase transcriptomes further away and lactose transcriptomes clustered closer together. Replicates of each sample clustered together except for sample replicates lac mid_01 and lac mid_02 which had a wider separation than other samples. This was shown in the R^2^ values in Fig. 3B with the samples having a correlation value of 0.820 compared to an average of 0.981 for the rest of the replicates (Fig. 3A). The 2′-FL samples averaged a R^2^ value of 0.862 compared to their subsequent control with the lowest value of 0.850 with lactose late-log vs 2′-FL late-log. The lowest 2′-FL samples correlation was between 2′-FL early and 2′-FL late (0.943) while 2′-FL early and 2′-FL mid-log had the highest values (0.985). Overall lactose values were above 0.955 for all the sample comparisons for that test type.

**Figure 4:**
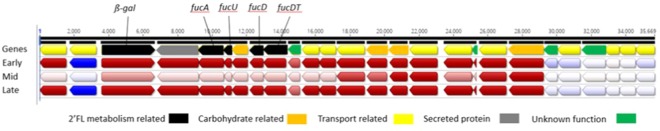
Expression levels of the HMO cluster found in *Bifidobacterium longum* subsp. *infantis* Bi-26. Genes are colored coded by their functionality. Early, mid, and late log phases show the expression levels of the cluster throughout 2′-FL fermentation. Red indicates upregulation in 2′-FL transcriptome samples, white same for both conditions, and blue is upregulation on lactose transcriptome samples. 2′-FL metabolism related genes (Table S2) are labeled according to gene name.

Only 18 out of the 25 genes in the Bi-26 HMO cluster had an increase in transcription when grown on 2′-FL (Fig. 4) compared to the lactose control. The seven genes that had equal or more expression in the lactose samples annotated as carbohydrate permease and ATP transporter genes. In total 244 genes were upregulated in 2′-FL fermentation (Supplementary Table 2) compared to the lactose control. Among them, several novel membrane transportation clusters throughout the genome were identified to have higher expression when grown on 2′-FL. Three genes clustered together, annotated as ABC-type sugar transport system periplasmic or permease components locations 1,901,517 through 1,904,967 for the 2′-FL early compared to lactose early phase. Values for these genes throughout the fermentation maintained a positive significant difference in expression. Another cluster containing 3 genes annotated as *msmF*, *msmG*, and “Multiple sugar ABC transporter, substrate-binding protein” showed upregulation for the 2′-FL early samples as well as being significantly upregulated throughout the entire growth cycle compared to the control.

**Figure 5:**
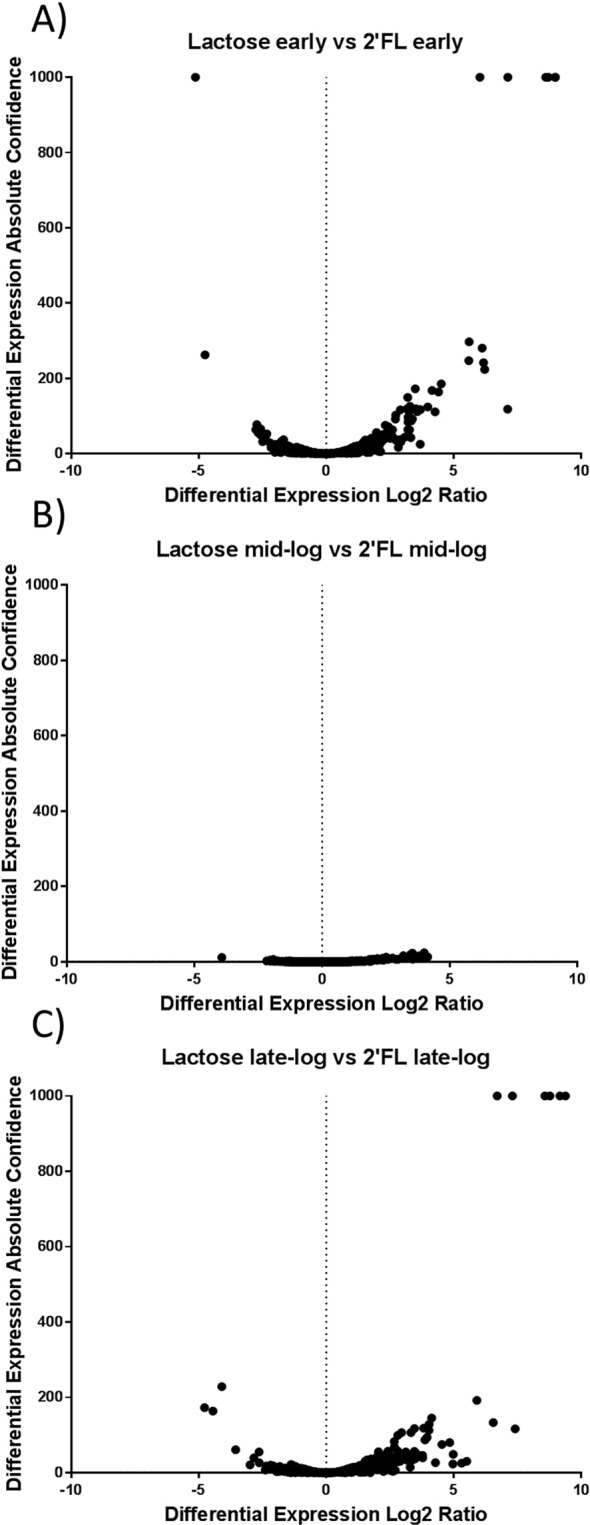
Volcano plots of differential expression compared to absolute confidence for 2′-FL RNA-seq values. 2′-FL is compared to lactose experiments in (**A**) early phase, (**B**) mid-log phase, and (**C**) late-log phase. Only values with a Log2 ratio above 1 and an absolute confidence above 1.30 (p-value 0.05) were considered significant.

Many other sugar transport systems had significant upregulation compared to the lactose control (Supplementary Table 3). When comparing transport throughout 2′-FL fermentation alone, one gene was significantly upregulated in the 2′-FL early samples compared to the 2′-FL mid-log samples (Supplementary Fig. 2). This gene was identified as a permease of the major facilitator family and was also more upregulated during late log phase than mid-log phase. A total of 29 permeases or transporters were upregulated in all three phases of 2′-FL samples while four unique transport genes were found in the early phase and 7 were found in the late phase only (Supplementary Table 3). Figure 5 displays the comparisons of the three measured stages in 2′-FL fermentation compared to the lactose control. 2′-FL resulted in 244 individual genes to be upregulated across all of the samples. Supplementary Fig. 2 shows the expression profile of the genes throughout the different phases of the fermentation. All the fucose related genes in Supplementary Table 2 were found to be upregulated in all samples except for the triose-phosphate isomerase *tpiA* gene which was never significantly upregulated. A summary of the expression of the genes of interest is shown in Supplementary Table 4. The formate *fpl* gene was upregulated in the mid-log and late-log phases only. None of the other genes listed in Supplementary Table 2 were upregulated in response to lactose or 2′-FL in any of the samples taken. Early and mid-log samples had a strong correlation (R^2^ value of 0.985) and only had one gene, related to sugar transport, that was significantly different between the two phases (Supplementary Fig. 2). Mid-log vs late-log samples had a total of 140 gene differences with late-log having 3 fucose genes upregulated compared to mid-log. These genes were *fucA*, *fucU* and *fucD*, but were not significantly upregulated compared to the early samples. Late-log samples also had 23 unique transport genes upregulated compared to the mid-log phase (Supplementary Table 3). Early and mid-log phase had 26 and 28 transport genes that were unique compared to late-log phase samples.

### Metabolite profile of 2′-FL fermentation in *B. longum* subsp. *infantis* Bi-26

**Figure 6:**
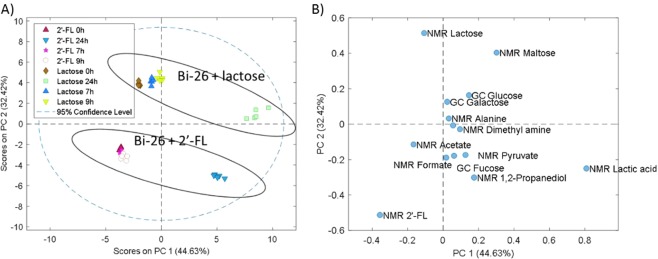
PCA score for individual samples (**A**) and loading plots of all quantified metabolites (**B**) from GCMS and NMR. Samples are described by the time that they were taken with 0h being the initial sample, 7h being early phase, 9h being mid-log and 24 h being late-log phase.

**Figure 7:**
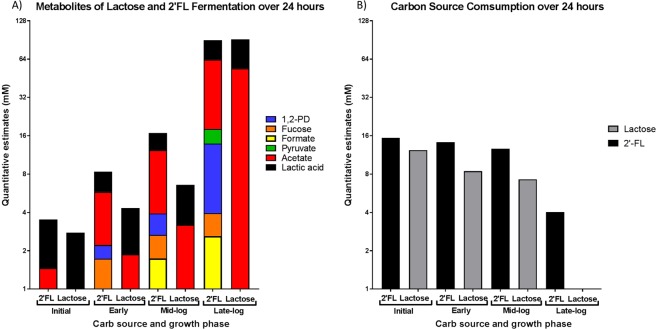
Metabolites of lactose and 2′-FL fermentation all phases of growth tested. (**A**) Levels of metabolites for 2′-FL or lactose fermentation. Each sample along with growth phase is listed below. (**B**) Levels of carbon source for each of the phases tested.

Samples of Bi-26 grown on 2′-FL and lactose were taken at early, mid-log, and late-log. Samples were quantified for metabolites using GCMS and NMR. The data were first analyzed using a PCA score plot and showed a clear grouping between the 2′-FL and lactose samples and a time effect for both treatments (Fig. 6). Figure 6 shows a clear separation of 2′-FL samples from lactose samples. This resulted in independent grouping of the lactose phases of growth and grouping of 2′-FL phases of growth. The late-log phases had both test types clustering closer to each other but still the groups clustered to its own carbon source. Showing that the late-log samples are much more unique than the earlier phases of growth. 2′-FL fermentation resulted in the formation of fucose, acetate, lactate, 1,2-PD and formate along with the utilization of 2′-FL (Fig. 7, Supplementary Table 5). 2′-FL was consumed in the early, mid-log and late-log phases of growth with 73.8% of the added 2′-FL consumed. Levels of fucose, lactic acid and acetate rose in all phases of growth. Levels of 1,2-PD increased over the entire growth period showing the ability of Bi-26 to break down all monomers of 2′-FL. Pyruvate was also detected at the late-log phase and was not detected in any of the other samples taken. Lactose fermentation resulted in the formation of lactate, acetate, and formate (Fig. 7, Supplementary Table 5). Lactose was completely consumed by the last sample taken while the production of 1,2-PD, fucose and formate were undetectable at the late-log phase. Lactic acid was also detected with levels rising throughout the 24-hour fermentation. Lactose fermentation resulted in 40.0% more lactic acid through late-log phase compared to 2′-FL. Lactose fermentation created 19.1% more acetate at the late-log phase but 2′-FL fermentation at the early and mid-log phases were 220% and 300% greater than lactose. Formate levels were higher in 2′-FL fermentation with concentrations being 370%, 440% higher in the early and mid-log phases of growth while formate was at undetectable levels by NMR spectroscopy in the lactose samples. Pyruvate was only detected in the late-log 2′-FL sample (4.23 mM) and was not detected in any of the lactose samples. 1,2-PD and fucose were also not detected in the lactose fermentation samples as can be seen on the compared spectra in Supplementary Fig. 3. Lactose consumption was at a consistent rate of around 0.57 mM/hr, while 2′-FL was varied (0–7 h = 0.167 mM/hr, 7–9 h = 0.79 mM/hr, 9–24 h = 0.57 mM/hr) (Supplementary Table 5). Bi-26 was able to initially convert 2′-FL to acetate and lactate at a higher rate than lactose. Over the last 15 hours of fermentation, lactose did surpass 2′-FL almost double per mM carbon source. Fucose production was highest in the early phase of the fermentation before decreasing in later samples.

## Discussion

*B. longum* subsp. *infantis* is one of the primary early colonizers of the neonate^25,26^ and can process 2′-FL, the most abundant oligosaccharide in human milk, into metabolites that feed other beneficial commensals^27,28^. This study is the first to combine RNA-seq and metabolomics to define the metabolism and metabolites of *B. longum* subsp. *infantis* Bi-26 fermentation of 2′-FL. Combining these sets of data, we were able to predict genes, detect gene transcription, and quantify the metabolites produced from the 2′-FL fermentation.

Bi-26 was able to ferment 2′-FL and lactose to a high cell density, consistent with the previously studied *Bifidobacterium longum* subsp. *infantis* strain ATCC 15697^19^. The two strains share many genes in the HMO utilization cluster and other necessary transport and enzymatic genes to break down each carbohydrate^12,19,29^. The RNA-seq data revealed several gene clusters involved in transport and utilization of 2′-FL that are also upregulated in the lactose control. Indeed, when fucose is cleaved from 2′-FL, the remaining lactose molecule is processed with the same genes as lactose alone. Furthermore, the data showed many genes besides the 2′-FL operon that were also involved in various stages of metabolism for the monomers of 2′-FL throughout the fermentation process. Metabolite analysis confirmed the ability of Bi-26 to produce the 1,2-PD, acetate, formate and lactate as primary byproducts. The RNA-seq data also revealed several novel gene clusters annotated as carbohydrate transporters that were upregulated in all portions of 2′-FL fermentation compared to the lactose control. Interestingly one of those genes (EE567_09960), an annotated MFS transporter gene, was the only gene that was upregulated in the early growth phase compared to the mid-log when grown on 2′-FL. The lack of upregulation in the mid-log phase could be explained by the available lactose and glucose from the hydrolysis of 2′-FL. The lack of upregulation in the central metabolism genes could be explained by both carbon sources sharing the same genes.

The main differences between the 2′-FL and lactose fermentations resulted from fucose metabolism and formate production, which were not detected in lactose samples at late-log. The fucose pathway is not well defined in bifidobacteria, as some studies suggest a phosphorylated pathway^12^ while others suggest non-phosphorylated pathway^18^. Our data coincides with Bunesova’s^18^ findings, since Bi-26 lacks the *fucose isomerase*, *fucose aldolase*, and *fucose kinase* genes required to perform phosphorylated metabolism of fucose. This is further supported by the upregulation of the fucose genes (Supplementary Table 2) and production of 1,2-PD. Gene expression also differs from some past studies^17,30^, as the eight genes in the HMO operon have the same or increased expression on lactose compared to 2′-FL. Two of those genes have unknown functions while five of them are transport related. This difference in regulation may be due to our use of purified 2′-FL compared to the complex mixture of HMOs that were previously studied^29^, which would require other transporters and enzymes from the HMO cluster for their metabolism. Further studies, such as knockout experiments, could be used to help define the specific genes needed for metabolism and transport of HMOs.

The metabolite data revealed that 2′-FL fermentation resulted in a higher overall diversity of metabolites and higher concentrations of formate, fucose, and 1,2-PD compared to the lactose controls. The higher proportions of acetate to lactate compared in the early to mid-log phases are similar to previous findings^30^. Higher levels of acetate and lactic acid per mM of substrate show the ability of Bi-26 to quickly change the surrounding environment even in a transient state of bacteria and carbon sources^31^. The increased levels of acetate and formate allow Bi-26 to balance its reduction-oxidation (redox) levels and produce enough energy to continue growth. In the case of 2′-FL, fucose fermentation results in the net loss of 1 NADH which has to be regenerated through other metabolic processes (Fig. 1). Through the production of formate, Bi-26 generates NADH from NAD^+^ without having to use NADH to reduce pyruvate to lactate, resulting in a net positive NADH (Fig. 1). The main gene involved in formate production was upregulated in the mid-log and late-log samples, suggesting NADH is only required after the cells start reproducing rapidly. This, combined with the low levels of 1,2-PD (<1 mMol) produced, shows preferential use of the glucose and galactose monomers of 2′-FL in early growth phase. The slower consumption rate of 2′-FL (−0.17 vs −0.56 mM/h) compared to lactose in the early phase suggests the additional time is necessary for enzyme production, transport, and processing of 2′-FL. To provide the necessary ATP for cell growth, both lactose and 2′-FL utilize the acetate pathway which produces more ATP than pyruvate and conserves NADH (Fig. 7, Table S4). The bifid shunt allows the bacteria to adapt to the current conditions by regenerating cofactors or producing ATP while producing SCFAs to benefit the host^27^. Also, this adds the ability to process more complex carbohydrates, such as 2′-FL and other HMOs by their ability to regenerate cofactors and balance redox through various metabolites using the bifid shunt.

*Bifidobacterium* utilization of fucosylated HMOs, such as 2′-FL, has been shown as a key colonization factor for infants^32^. Since 2′-FL is not broken down by the host^33^, 2′-FL would be available for fermentation by the gut microbiome. *B. longum* subsp. *infantis* has prolonged colonization in breast-fed infants linked to HMOs^25^. Furthermore, the concentrations of lactate and acetate were significantly higher and fecal pH was lower in *B. longum* subsp. *infantis*-fed infants compared to the control group without probiotic supplementation^25^. Higher levels of acetate protect the human gut from infection^34^, reduce inflammation^35^ and provide a positive immune response^36^. Formate and 1,2-PD are beneficial in the gut by inhibiting pathogens or as substrates for beneficial bacteria^34^. Lactate and short-chain fatty acids, such as formate and acetate, as well as other metabolites create an acidic environment favoring the growth of other bifidobacteria and commensals over potential pathogenic bacteria^5,25,37^. For example, *E. hallii*, can convert 1,2-PD to propionate that has potential health promoting impact on host gut epithelium, immune system and gluconeogenesis in the liver^27,38–40^. Looking into these relationships could potentially show how bacteria work together in the newly colonized gut of the infant.

With these results we are able to show the products of 2′-FL fermentation by *B. longum* subsp. *infantis* Bi-26 and connect those products to the genes that are being transcribed to create them. By using these strategies to identify key genes outside of typical operons, we can better understand the metabolism of 2′-FL and other HMOs. Our study supports the potential synergetic benefits of Bi-26 and 2′-FL as a supplement for infants to maintain a favorable acidic gut environment.

## Materials and Methods

### Bacterial cultures

Vials of *Bifidobacterium longum* subsp. *infantis* strain Bi-26 (ATCC SD-6720; Bi-26) culture were obtained from the internal DuPont culture collection. Bi-26 was grown under anerobic conditions using modified *Bifidobacterium* medium 58 (mBM58) at 37 °C. mBM58 from Deutsche Sammlung von Microorganismen und Zellculturen; [casein peptone, tryptic digest 10 g/L, yeast extract 5 g/L, meat extract 5 g/L, K_2_HPO_4_ 3 g/L, ascorbic acid 10 g/L, and cysteine-HCl 0.5 g/L; pH 6.8]. Stock suspensions (20% w/v) of 2′-FL (DuPont N&H/Inbiose, Ghent, Belgium), and lactose (Sigma-Aldrich, St. Louis, MO, USA) were prepared in MQ-water and sterile filtered (0.2 µm Minisart®, Sartorius AG, Göttingen, Germany).

### Genome sequencing

Bi-26 was sequenced using Illumina MiSeq.

#### Genomic DNA isolation

Genomic DNA for sequencing was prepared by using the MasterPure^TM^ Complete DNA and RNA isolation kit (Illumina, Madison, Wi.) per the manufacturer’s directions on an overnight culture of Bi-26 grown in mBM58 broth.

#### MiSeq sequencing

Libraries were prepared with the Hyper Library construction kit (Kapa Biosystems), pooled, and quantitated by qPCR prior to sequencing on two flowcells for 251 cycles using paired-end 250 bp MiSeq V3 bulk sequencing kit (Illumina, version 3). Fastq files were generated and demultiplexed with bcl2fastq v2.17.1.14 Conversion Software.

#### Sequence assembly and annotation

Fastq reads were quality trimmed using sickle (v 1.33; 1)^41^ and assembled using the SPAdes^42^ de-novo genome assembler (v3.9.1; 2) under standard default conditions. Completed draft was uploaded to Rapid Annotation by Sub-System Technology (RAST) using the default conditions for annotation^43–45^.

#### Comparative genomics

Genomes were aligned using progressive Mauve in Windows^46^. Genes of interest and segments of the genome were aligned using Geneious v 11.0.4 (Biomatters, Auckland, New Zealand). Genomic features and analysis (Supplementary Table 1) were generated from RAST. Presence of 2′-FL related genes was determined by searching the genomes for specified enzyme class (EC) number using Geneious (Supplementary Table 2). Average nucleotide identity was determined using pyani^47^.

### Growth curves

Bi-26 was grown overnight (18 hrs) in mBM58 supplemented with 1% lactose. 50 uL of this was used as an inoculum in 5 mL of mBM58 with either 1% w/v 2′-FL, lactose or no carbohydrate added. Lactose was used as the positive control while no carbohydrate added was used as a negative control. Cultures were initially tested for absorbance at 600 nm (A600) then tested at time points 2, 4, 5, 6, 8, 12 and 24 hours. The initial zero value was set after inoculation. All fermentations took place under anaerobic conditions and at 37 °C. Graphs and analysis were performed in PRISM v7 (GraphPad, La Jolla, CA).

### RNA-sequencing

#### Samples

Overnight culture of Bi-26 in mBM58+ lactose was used as a 1% inoculum for 3 tubes of 10 mL of either mBM58+ 2′-FL or mBM58+ lactose. Lactose was used as the control samples. A600 was checked according to the growth curve and samples (one 10 mL tube) were taken during early phase (A600 = 0.25–0.30), mid log (A600 = 0.5–0.7), and late log (A600 = 0.9–1.1).

#### RNA extraction

Immediately after each sample reached the growth phase, samples were centrifuged at 10,000 x g for 5 minutes, supernatant was removed, and the pellet was re-suspended in 1 mL of Trizol and frozen at −70 °C. The cell pellets were later thawed, transferred to a Lysing Matrix B 2 mL tube (MPBio, 116911050), and disrupted using a Bead ruptor elite (OMNI international, Georgia, United States) at 6.30 m/s for 2 minutes. The lysate was subjected to a chloroform organic extraction and followed by purification using RNeasy Mini Kit (Qiagen, Hilden, Germany, 74104).

#### Sequencing

Ribosomal RNA was removed prior to library construction using Ribo-Zero rRNA Removal Kit Gram-Positive Bacteria (Illumina, MRZGP126). Stranded cDNA libraries were prepared using TruSeq Stranded mRNA Kit (Illumina, 20020594), quantitated by Agilent TapeStation, pooled, and sequenced on one flowcell lane for 75 cycles using paired-end 75 basepair sequencing on Illumina 2500 HiSeq Rapid Cluster Kit (Illumina, V2, PE-402-4002) and HiSeq Rapid SBS Kit (Illumina, V2, FC-402-4021).

#### RNA-seq assembly

Paired-end reads were imported and mapped to the Bi-26 genome using the RNA mapping tool SeqMan Pro in DNAStar V12.3.1.4 (Madison, Wi) under the default settings. Processed using QSeq, and normalized by reads per kilobase of transcript per million mapped reads (RPKM).

#### Statistics

Regression analyses of the RNA data were made in ArrayStar software V12.3.1. Statistical analyses between sets of samples were analyzed using DESeq 2 method in Geneious 11.0.4. Differences in expression were considered significant if the Absolute Confidence (−Log10 adjusted p-value) was +1.00, representing, and the Log_2_ ratio was at +1.00, representing p < 0.05 and a >2x fold change, respectively, after normalization.

### Metabolite analysis

#### Sample preparation

Bi-26 was first pre-cultivated anaerobically overnight at 37 °C using mBM58. For metabolite analysis, Bi-26 was grown at total volume of 60 ml with either 2′-FL or lactose as carbon source (1% w/v in the final concentration) in mBM58. Five biological replicates were performed for the metabolite analysis. Samples were collected from growth vessels at initial time 0, early phase (7 hours), mid-log phase (9 hours) and late-log phase (24 hours) of growth and centrifuged (Biofuge Stratos, Heraeus Instruments, Osterode, Germany) to remove bacteria. Supernatants were then sterile filtered (0.2 µm Acrodisc® syringe filters, Pall Life Sciences, Ann Abor, MI, USA) and divided to aliquots of ca. 0.8 mL. Samples were stored frozen at −20 °C.

#### GCMS analysis

Reagents and solvents for the GC/MS analysis were pyridine and N-methyl-N-trimethy- lsilyl-trifluoroacetamide (MSTFA) from Fisher Scientific (Hampton, NH, USA) as well as trimethylchlorosilane (TMCS) Sigma-Aldrich (St. Louis, MO, USA). Internal standards included sorbitol-^13^C_6_ Sigma-Aldrich (St. Louis, MO, USA) and in addition heptadecane and norvaline (Fisher Scientific, Hampton, NH, USA). Standards for absolute quantification were 1,2-PD, fucose, glucose, lactic acid, lactose monohydrate and pyruvic acid from Sigma-Aldrich (St. Louis, MO, USA), galactose (Fisher Scientific, Hampton, NH, USA) and 2′-FL (CarboSynth Compton, United Kingdom). Methoximation reagent was prepared by weigh-in of 0.5 g methoxyamine hydrochloride, Sigma-Aldrich (St. Louis, MO, USA), and 10 mL pyridine was added. Silylation reagent was prepared by adding 100 µL TMCS to 9.9 mL MSTFA. An aliquot of 100 µL sample was dried at 40 °C/vacuum overnight together with norvaline (approx. 20 µg) added as internal standard. Methoximation was performed on all samples as a batch process by addition of 100 µL methoximation reagent and reaction for 90 min at 37 °C, and 750 RPM on a Heidolph Vibramax 100 (Heidolph Instruments GmbH & Co. KG, Schwabach, Germany). Subsequently, 10 µL internal standard stock solution, containing sorbitol-^13^C_6_ (approx. 70 µg) and heptadecane (approx. 90 µg) was added, using the multipurpose sampler. Then, silylation (37 °C/30 min) was performed just-in-time by addition of 250 µL silylation reagent. After silylation 200 µL of pyridine is added before the GC-injection by the Gerstel Multipurpose MPS2 sampler. A pooled sample produced by sampling an aliquot from all samples in the set was analyzed a number of times with the samples in parallel to a number of blank samples. Each sample was analyzed in triplicate. All vials were analyzed in random order. For the determination of responses factors relative to sorbitol-^13^C_6_ ISTD, thus for absolute quantification, aqueous solutions of 1,2-PD, fucose, galactose, glucose, lactose, lactic acid, pyruvic acid and 2′-FL in six dilution levels were analyzed as samples. All data acquisition was performed on a system consisting of an Agilent 7890A gas chromatograph (Agilent Technologies, Santa Clara, CA) with a Gerstel Multipurpose MPS2 autosampler (Gerstel, Inc., Linthicum, MD) interfaced to a LECO Pegasus time-of-flight mass spectrometer with an electron ionization (EI) source (LECO Corporation, St. Joseph, MI). The GC was mounted with 30 m × 0.25 mmID × 0.25 µm 5%Phenyl-95%methyl-silicone capillary column, RTX5 (Restek, Bellefonte, PA) with a 0.5 m similar precolumn. Injection was 1 µL with a split ratio of 1:20 in a split/splitless injector kept at 280 °C mounted with an Agilent Deactivated Split Taper Inlet Liner. The column was operated with a helium flow of constant ca. 1 ml/min, fine adjusted to maintain retention time for three internal standards within ±0.5 seconds. The transfer line was maintained at 250 °C, and the oven temperature ramp initial 50 °C, followed by 10 °C/min to 320 °C, which is then kept for 10 minutes. The MS conditions were with −70 eV electron energy, ion source temperature of 250 °C, acquisition delay 180 seconds, acquisition rate 20 spectra/s and a mass range of *m/z* 70–1000. The data processing was performed using LECO ChromaTOF v.4.71.0.0 and GeneData Expressionist Refiner and Analyst version 10.5 (GeneData AG, Basel, Switzerland) LECO data files were loaded to GeneData Expressionist Refiner and individual isotopic masses were summed to nominal masses and subjected to a series of noise reduction including smoothing and signal intensity clipping steps. Peaks were detected and grouped and were assignment of based on the AMDIS algorithm towards an in-house spectral library. Relative comparisons of profiles were based on responses calculated as a characteristic ion for the compound divided with a characteristic ion for the sorbitol-^13^C_6_ internal standard. Absolute quantification was performed for a selected number of compounds based on response factors determined with authentic standards relative to the sorbitol-^13^C_6_ ISTD.

#### NMR spectroscopy

Sample preparation for NMR spectroscopy were performed by mixing 150 µL Bi-26 fermentation with 30 µL of D_2_O containing 0.05% (w/v) trimethylasilylpropionic acid sodium salt (TSP) and transferring the sample to a 3 mm NMR tube. NMR measurements were performed at 298 K on a 600 MHz Bruker Ascend spectrometer (Bruker Biospins, Rheinstetten, Germany) operating at 14.1T and equipped with a 5 mm triple resonance (TXI) probe. ^1^H NMR spectra were acquired using a standard 1D noesy experiment (noesypr1d, Bruker pulse sequence) including water presaturation. The spectra were acquired by 64 scans, 64 k data points, spectral width of 11.97 ppm, an acquisition time of 4.56 s and a recycle time of 3 s. The Free Induction Decay (FID) obtained was multiplied by 0.8 Hz of exponential line broadening before Fourier transformation. In addition, 2D ^1^H-^13^C heteronuclear single quantum coherence (HSQC; hsqcedetgpsisp2.3, Bruker pulse sequence) experiments were acquired with spectral width of 12.02 ppm in the ^1^H dimension and 165 ppm in the ^13^C dimension, a data matrix with a size of 2048 × 256 data points, 32 transients per increment and a recycle delay of 2 s. The spectra were referenced to TSP (chemical shift 0 ppm), phased and baseline corrected in Topspin 3.0 software (Bruker, Rheinstetten, Germany). The NMR spectra were imported into Matlab version 2017a (The MathWorks, Inc., Natick, MA, USA), aligned using icoshift version 1.3.1 (Savorani 2010) and binned according to an optimized bucketing algorithm (Sousa 2013). Quantification of selected metabolites was performed by manual integration of TSP (0 ppm (s); intern standard), valine (1.03 ppm (d)), 1,2-PD (1.13 ppm (d)), fucose (1.19 ppm (d)), 2′-FL (1.24 ppm (d)), lactate (1.33 ppm (d)), alanine (1.47 ppm (d)), acetate (1.93 ppm (s); 2.04 ppm (s) at 24 h samples), pyruvate (2.36 ppm (s)), dimethyl amine (259 ppm (s)), maltose (3.40 ppm (dd)), lactose (4.68 ppm (d)), glucose (5.23 ppm (d)) and formate (8.45 ppm (s)). A representative ^1^H NMR spectrum of a 24h 2′-FL sample can be seen in Supplementary Fig. 3.

#### Metabolite statistics

Principal component analysis (PCA) and orthogonal partial least squares discriminant analysis (OPLS-DA) using PLS Toolbox (eigenvector Research, U.S.A.) were done on both Pareto scaled GCMS and NMR-data to detect clustering behavior and elucidate biochemical differences between pre-defined classes, respectively.

## Supplementary information

Supplementary information

## Data Availability

*B. longum* subsp. *infantis* Bi-26 is safe deposited as ATCC SD-6720. The genome sequence for *B. longum* subsp. *infantis* Bi-26 is available at the National Center for Biotechnology Information under accession number RJJM00000000. The expression data is available with the Gene Expression Omnibus number GSE122350.
