# Supplementary material for: Novel Genes and Metabolite Trends in Bifidobacterium longum subsp. infantis Bi-26 Metabolism of Human Milk Oligosaccharide 2′-fucosyllactose
Source: Sci Rep. 2019 May 28;9:7983. doi: 10.1038/s41598-019-43780-9 (PMC6538704; doi:10.1038/s41598-019-43780-9)
Supplement: Supplementary file 1 — Supplementary information [file 41598_2019_43780_MOESM1_ESM.pdf]

SUPPLEMENTAL MATERIAL

Supplementary Figures 1-3 and Supplementary Tables 1-5 for manuscript:

Novel Genes and Metabolite Trends in *Bifidobacterium longum* subsp. *infantis* Bi-26 Metabolism of Human Milk Oligosaccharide 2'-fucosyllactose

Bryan Zabel[1], Christian Clement Yde,[2] Paige Roos,[3] Jørn Marcussen,[2] Henrik Max Jensen,[2] Krista Salli[4], Johanna Hirvonen[4], Arthur C. Ouwehand[4], Wesley Morovic[1]

[1]Genomics & Microbiome Science, DuPont Nutrition & Health, Madison, WI, USA
[2]Advanced Analysis, DuPont Nutrition Biosciences ApS, Brabrand, Aarhus, Denmark

[3]Genomics Lab, DuPont Pioneer, Johnston, IA, USA

[4]Global Health and Nutrition Science, DuPont Nutrition and Health, Kantvik, Finland

General genome feature	***B. longum subsp. Infantis* strain Bi-26**	***B. longum subsp. Infantis* strain ATCC15697**
Size	2,569,657	2,832,748
GC content	59.4%	59.9%
Predicted coding density	84.7%	87.3%
number of subsystems	286	284
number of coding sequences	2280	2637
number of RNAs	60	91
**Subsystem features**		
Amino Acids and Derivatives	201	201
Carbohydrates	146	145
Cell Division and Cell Cycle	19	19
Cell Wall and Capsule	61	56
Cofactors, Vitamins, Prosthetic Groups, Pigments	103	104
DNA Metabolism	63	57
Dormancy and Sporulation	2	2
Fatty Acids, Lipids, and Isoprenoids	35	33
Iron acquisition and metabolism	1	5
Membrane Transport	35	34
Metabolism of Aromatic Compounds	3	3
Plant	8	8
Miscellaneous	4	4
Motility and Chemotaxis	2	0
Nitrogen Metabolism	7	7
Nucleosides and Nucleotides	57	56
Phages, Prophages, Transposable elements, Plasmids	8	13
Phosphorus Metabolism	27	27
Potassium metabolism	4	4
Protein Metabolism	192	201
Regulation and Cell signaling	16	16
Respiration	2	2
RNA Metabolism	58	58
Secondary Metabolism	2	0
Stress Response	31	32
Sulfur Metabolism	9	9
Virulence, Disease and Defense	32	33

Supplementary Table 1: General genome feature comparison of *Bifidobacterium longum* subsp. *infantis* Bi-26 to *Bifidobacterium longum* subsp. *infantis* strain ATCC15697. Genomic assembly statistics along with subsystems (annotated from RAST) are displayed.

			Presence in B. longum subsp. infantis genomes								
**Leloir pathway genes**	**Gene name**	**Enzyme**	**Bi-26**	**ATCC15697**	**BT1**	**Bifido_12**	**TPY12-1**	**IN-07**	**Bifido_04**	**IN-F29**	**EK1**
galactokinase	*galK*	EC 2.7.7.6	+	+	+	+	+	+	+	+	+
UDP-glucose 4-epimerase	*galE*	EC 5.1.3.2	+	+	+	+	+	+	+	+	+
galactose-1-phosphate uridylyltransferase	*galT*	EC 2.7.7.10	+	+	+	+	+	+	+	+	+
phosphoglucomutase	*pgm*	EC 5.4.2.2	+	+	+	+	+	+	+	+	+
**Main Fermentative pathway genes**											
glucokinase glkA Blon_0565	*glkA*	EC 2.7.1.63	+	+	+	+	+	+	+	+	+
glucose-6-phosphate isomerase	*gpi*	EC 5.3.1.9	+	+	+	+	+	+	+	+	+
fructose-6-phosphate phosphoketolase	*xfp*	EC 2.6.1.16	+	+	+	+	+	+	+	+	+
transaldolase	*tal*	EC 2.2.1.2	+	+	+	+	+	+	+	+	+
transketolase	*tkt*	EC 2.2.1.1	+	+	+	+	+	+	+	+	+
ribose 5-phosphate isomerase	*rpiA*	EC 5.3.1.6	+	+	+	+	+	+	+	+	+
Ribulose-phosphate 3-epimerase	*rpe*	EC 5.1.3.1	+	+	+	+	+	+	+	+	+
xylulose-5-phosphate phosphoketolase	*xfp*	EC 4.1.2.9	+	+	+	+	+	+	+	+	+
glyceraldehyde-3-phosphate dehydrogenase	*gap*	EC 1.2.1.12	+	+	+	+	+	+	+	+	+
phosphoglycerate kinase	*pgk*	EC 2.7.2.3	+	+	+	+	+	+	+	+	+
phosphoglycerate mutase	*pmg*	EC 5.4.2.1	+	+	+	+	+	+	+	+	+
enolase	*eno*	EC 4.2.1.11	+	+	+	+	+	+	+	+	+
pyruvate kinase	*pyk*	EC 2.7.1.40	+	+	+	+	+	+	+	+	+
lactate dehydrogenase	*idh*	EC 1.1.1.27	+	+	+	+	+	+	+	+	+
**Fucose genes**											
Alpha-L-fucosidase	*fucA*	EC 3.2.1.51	+	+	+	+	+	+	+	+	+
Fucose permease	*fucP*	NA	+	+	+	+	+	+	+	+	+
dehydrogenase clustered with L-fuconate utilization genes	*NA*	EC 1.1.1.122	+	+	+	+	+	+	+	+	+
L-fuconate dehydratase		EC 4.2.1.68	+	+	+	+	+	+	+	+	+
L-fucose mutarotase	*fucU*	EC 5.1.3.29	+	+	+	+	+	+	+	+	+
lactaldehyde reductase	*fucO*	EC 1.1.1.77	+	+	+	+	+	+	+	+	+
triose-phosphate isomerase	*tpiA*	EC 5.3.1.1	+	+	+	+	+	+	+	+	+
**Acetate gene**											
acetate kinase	*ackA*	EC 2.7.2.1	+	+	+	+	+	+	+	+	+
**Formate gene**											
pyruvate formate-lyase	*pfl*	EC 2.3.1.54	+	+	+	+	+	+	+	+	+

Supplementary Table 2: Annotated genes related to 2'-FL utilization in Bi-26 and other *B. longum* subsp. *infantis* strains. Used in conjunction with Figure 1 to describe genes needed to make quantified metabolites. Included are gene name, KEGG enzyme class and pathway the gene is located in. Presence in other *B. longum* subsp. *infantis* strains is noted. Presence of genes was determined by searching for enzyme class number or annotations using Geneious. The strains selected were those that clustered with the type strain ATCC15697 in the National Center for Biotechnology Information (NCBI) *B. longum* dendrogram located at https://www.ncbi.nlm.nih.gov/genome/183? Accessed 04/21/2019.

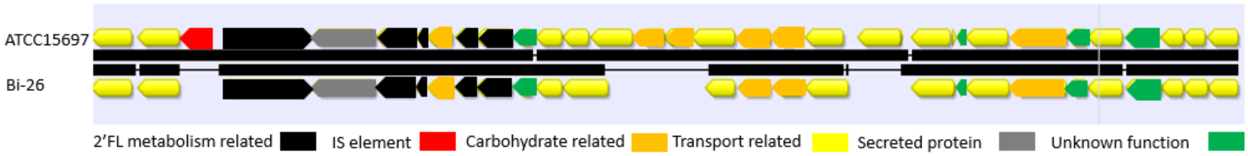

Supplementary Figure 1: Alignments of ATCC15697 and Bi-26 HMO cluster using Geneious. Genes are colored by their functionality. The middle black section of the figure shows the mismatches in translation. Gaps in Bi-26 for the IS element, transport proteins and secreted protein are where the genes are not present.

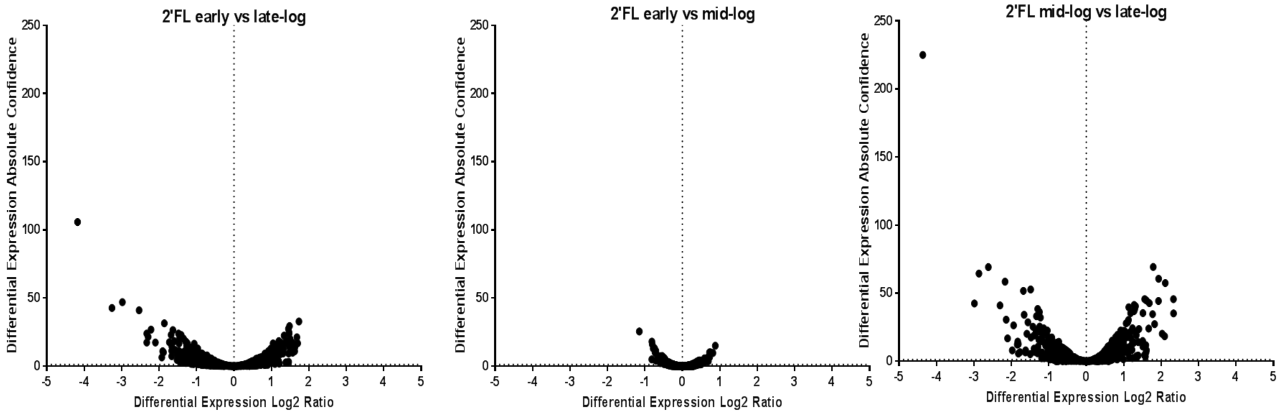

Supplementary Figure 2: Volcano plots of differential expression compared to absolute confidence for 2'-FL RNA-seq values compared to the other phases of growth on 2'-FL. Only values with a Log2 ratio above 1 and an absolute confidence above 1.30 (p-value 0.05) were considered significant.

A)

Phase(s)	Fucose related	Hypothetical	Transport related	Central metabolism	Other	Total
All 3	9	14	29	31	12	95
Early only	0	8	4	13	4	29
Mid-log only	0	7	0	0	1	8
Late-log only	0	15	7	28	5	55
Early and mid	0	2	1	3	0	6
Early and late	0	16	4	19	8	47
Mid and late	0	1	1	2	0	4

B)

Comparison	Early vs mid		Early vs late		Mid vs late	
Phase	Early	Mid-log	Early	Late	Mid-log	Late
Fucose related	0	0	0	0	0	3
Hypothetical	0	0	29	11	24	17
Transport related	1	0	26	16	28	23
Central Metabolism	0	0	25	18	14	12
Other	0	0	14	15	7	12
Total	1	0	94	60	73	67

Supplementary Table 3: (A) Comparison of genes functions upregulated in 2'-FL fermentation and at what part of the fermentation they were upregulated in. (B) Comparing 2'-FL early vs mid and late and 2'-FL mid vs late for gene differences. To look at the differences in major systems throughout fermentation.

Genes/ Functions	Log2 Ratio early	p-value early	Log2 Ratio mid	p-value mid	Log2 Ratio late	p-value late
**Lactose Gene**						
Beta-galactosidase (EC 3.2.1.23) CDS HMO cluster	1.76363239	2.23E-42	1.453036987	1.11E-05	1.521517602	2.05E-14
**Leloir pathway genes**						
Galactokinase (EC 2.7.1.6) CDS	0.126435896	0.288887344	0.750285825	0.062522351	0.544604571	3.85E-05
UDP-glucose 4-epimerase (EC 5.1.3.2) CDS	0.810049707	1.89E-10	0.45840775	0.145733665	0.372488235	0.005699352
Galactose-1-phosphate uridylyltransferase (EC 2.7.7.10) CDS	1.099644411	8.78E-21	0.823699827	0.030696562	0.687430616	4.70E-08
Phosphoglucomutase (EC 5.4.2.2) CDS	-0.576976916	9.06E-06	0.181997756	0.567249427	0.11699882	0.3739832
**Main fermentation genes**						
Polyphosphate glucokinase (EC 2.7.1.63) CDS	0.655557986	5.34E-08	0.762651769	0.047869446	0.860681059	2.22924E-09
Glucose-6-phosphate isomerase (EC 5.3.1.9) CDS	0.901957471	6.90E-13	0.933528033	0.041092786	0.385425846	0.008517778
Fructose-6-phosphate phosphoketolase (EC 4.1.2.22) CDS	0.391267047	0.001118049	0.670967696	0.114195627	0.30370557	0.017148752
Transaldolase (EC 2.2.1.2) CDS	0.554885296	2.25E-06	0.756569661	0.08854475	-0.023665187	0.850368066
Transketolase (EC 2.2.1.1) CDS	0.191512513	0.14128884	0.488204592	0.182984772	0.220792855	0.102939386
Ribose 5-phosphate isomerase A (EC 5.3.1.6) CDS	0.383782011	0.006770406	0.861195531	0.050840225	0.572597746	7.65931E-05
Ribulose-phosphate 3-epimerase (EC 5.1.3.1) CDS	0.413140195	0.001308143	0.205487583	0.555270292	0.113840979	0.37594078
NAD-dependent glyceraldehyde-3-phosphate dehydrogenase (EC 1.2.1.12) CDS	0.365239397	0.003791971	0.577401413	0.116716001	0.122090122	0.308817639
Phosphoglycerate kinase (EC 2.7.2.3) CDS	0.47453399	4.62E-05	0.594644044	0.089754822	0.444910069	0.001448169
Phosphoglycerate mutase (EC 5.4.2.1) CDS	0.287742191	0.034010109	0.319510881	0.337332752	0.140411506	0.307780837
Enolase (EC 4.2.1.11) CDS	0.412183735	0.000482006	0.67203813	0.112037536	0.072738769	0.58552877
Pyruvate kinase (EC 2.7.1.40) CDS	0.155276824	0.194011771	0.1794255	0.575430108	-0.03776668	0.796051926
L-lactate dehydrogenase (EC 1.1.1.27) CDS	0.651064527	7.94E-07	0.596209199	0.130691092	-0.102965481	0.468593789
**Fucose genes**						
Alpha-L-fucosidase (EC 3.2.1.51) CDS Non hmo cluster	3.687712028	6.66E-27	2.157112872	8.01E-07	4.281550579	9.41698E-29
Alpha-L-fucosidase (EC 3.2.1.51) CDS HMO cluster	1.32547242	2.89E-24	1.453024153	0.001622438	1.572076226	7.27592E-27
dehydrogenase clustered with L-fuconate utilization genes CDS	8.708998093	0	3.625277007	1.1182E-12	9.163268073	0
dehydrogenase clustered with L-fuconate utilization genes CDS	1.505194366	1.34E-33	1.67300627	0.000363805	2.013244319	4.81E-47
L-fuconate dehydratase (EC 4.2.1.68) CDS	7.118010773	0	4.00346757	2.14E-28	7.302836379	0
L-fuconate dehydratase (EC 4.2.1.68) CDS HMO cluster	6.113032129	2.20E-283	1.692842419	0.000300618	2.048002117	1.36E-48
L-fucose mutarotase CDS	6.113032129	2.20E-283	3.541064248	6.06E-27	6.546959726	5.27E-136
L-fucose mutarotase CDS HMO cluster	1.436944157	3.46E-22	1.597622702	0.000962691	1.701493459	1.47E-21
Lactaldehyde reductase (EC 1.1.1.77) CDS	1.439913964	2.10E-27	1.849089764	1.28E-08	2.530493203	4.72E-40
Triosephosphate isomerase (EC 5.3.1.1) CDS	0.603615877	3.19E-06	0.523593576	0.108366676	0.748135852	4.60E-09
**Acetate gene**						
Acetate kinase (EC 2.7.2.1) CDS	0.393547168	0.000835347	0.731456777	0.101646728	0.29486821	0.026206859
**Formate gene**						
Pyruvate formate-lyase (EC 2.3.1.54) CDS	1.492721613	3.09E-35	1.127364895	0.000408604	1.423781291	4.23E-19

Supplementary Table 4: RNA-seq results of genes of interest in 2’-FL metabolism. Genes from Supplementary Table 2 log2 expression and p-value results from the early, mid-log and late phases are shown. Results are significant with a log2 of greater than 1 for 2’-FL samples and less than -1 for the lactose control samples with a p-value of less than 0.05. Green results are showing higher expression in 2’-FL samples while orange, yellow and red show higher expression in lactose samples or no change in expression.

Metabolite	Carb Source (mM)		Lactic acid (mM)		Acetate (mM)		Formate (mM)		Pyruvate (mM)		1,2-PD (mM)		Fucose (mM)	
Phase	2'FL	Lactose	2'FL	Lactose	2'FL	Lactose	2'FL	Lactose	2'FL	Lactose	2'FL	Lactose	2'FL	Lactose
Initial	15.40	12.33	2.06	1.97	0.67	0.63	0.16	0.17	ND	ND	0.15	ND	0.49	ND
Early	14.23	8.43	2.59	2.49	3.58	1.61	1.01	0.27	ND	ND	0.48	ND	0.72	ND
Mid-log	12.65	7.27	4.48	3.59	8.43	2.81	1.73	0.39	ND	ND	1.27	ND	0.92	ND
Late-log	4.03	ND	26.57	37.17	45.39	54.06	2.61	ND	4.23	ND	9.94	ND	1.34	ND

			Rate of production											
Metabolite	Carb Source (mM)		Lactic acid (mM)		Acetate (mM)		Formate (mM)		Pyruvate (mM)		1,2-PD (mM)		Fucose (mM)	
Phase	2'FL	Lactose	2'FL	Lactose	2'FL	Lactose	2'FL	Lactose	2'FL	Lactose	2'FL	Lactose	2'FL	Lactose
I→E	-0.17*	-0.56	0.08	0.07	0.42*	0.14	0.12*	0.01	NA	NA	0.05*	NA	0.03*	NA
E→M	-0.79*	-0.58	0.95*	0.55	2.43*	0.60	0.36*	0.06	NA	NA	0.40*	NA	0.10*	NA
M→L	-0.57*	NA	1.47*	2.24	2.46	3.42	0.06*	NA	NA	NA	0.58*	NA	0.03*	NA

			Rate of production per mM of carbon source											
Metabolite	Carb comsumed (mM)		Lactic acid (mM)		Acetate (mM)		Formate (mM)		Pyruvate (mM)		1,2-PD (mM)		Fucose (mM)	
Phase	2'FL	Lactose	2'FL	Lactose	2'FL	Lactose	2'FL	Lactose	2'FL	Lactose	2'FL	Lactose	2'FL	Lactose
I→E	1.17*	3.9	0.45*	0.13	2.49*	0.25	0.73*	0.03	NA	NA	0.28*	NA	0.20*	NA
E→M	1.58	1.16	1.20	0.95	3.07*	1.03	0.46*	0.10	NA	NA	0.50*	NA	0.13*	NA
M→L	8.62	7.29	2.56*	4.61	4.29*	7.03	0.10*	NA	0.49*	NA	1.01*	NA	0.05*	NA

*Significant difference compared to control

Supplementary Table 5: Overall metabolite results of each of the tested metabolites along with the carbon source are listed in the top table in mM values. The tables below contain the rates of production or consumption of each of the tested compounds. The middle table is valued with production over time while the lower table is valued at production per mM of carbon source consumed. Phases are labeled I=initial, E=early, M=mid-log, and L=late-log phases for the last two tables.

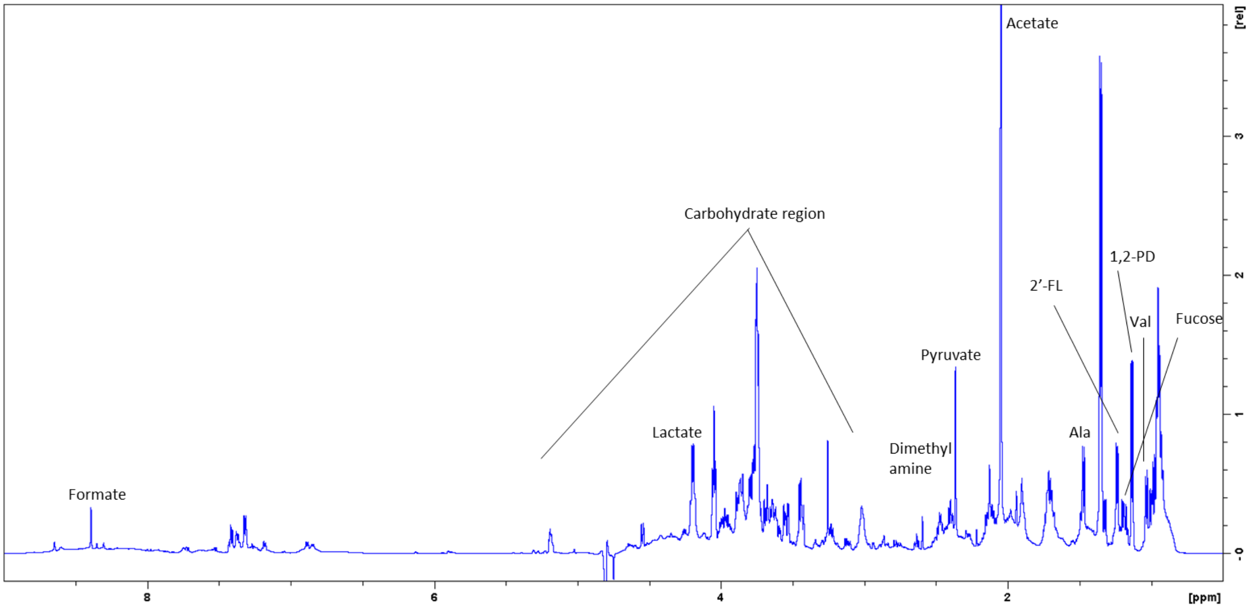

Supplementary Figure 3: Representative ^{1}H NMR spectra of a 24h 2'-FL fermentation sample. Selected quantified metabolites present at 24h 2'-FL fermentation have been labeled.
